# Handwriting measures as reflectors of executive functions among adults with Developmental Coordination Disorders (DCD)

**DOI:** 10.3389/fpsyg.2013.00357

**Published:** 2013-06-26

**Authors:** Sara Rosenblum

**Affiliations:** The Laboratory of Complex Human Activity and Participation, Department of Occupational Therapy, Faculty of Social Welfare and Health Sciences, University of HaifaHaifa, Israel

**Keywords:** handwriting, digitizer, executive functions

## Abstract

Planning ahead and organizational abilities in time and space are ingredients of high-level cognitive functions labeled as ‘Executive Functions’ (EF) required for daily activities such as writing or home management. EF deficits are considered a possible underlying brain mechanism involved in Developmental Coordination Disorders (DCD).

**The aim:** of the study was to compare the handwriting process measures and the planning and organizational abilities in space and time of students with DCD with those of matched controls and to find whether handwriting measures can predict daily planning and organizational abilities among students with DCD.

**Method:** 30 students diagnosed with DCD, between the ages of 24–41, and 30 age- and gender-matched controls participated in the study. They filled out the Handwriting Proficiency Screening Questionnaire (HPSQ) and the Adult Developmental Co-ordination Disorders Checklist (ADC). Furthermore, they copied a paragraph on a digitizer that is part of a computerized system Computerised Penmanship Evaluation Toll (ComPET).

**Results:** Significant group differences were found for the HPSQ subscales scores as well as for the temporal and spatial measures of the paragraph copy task. Significant group differences were also found for the planning and organizational abilities in space and time as reflected through the ADC subscales. Significant medium correlations were found in both groups between the mean HPSQ time subscale and the ADC-B subscale mean score (*r* = 0.50/0.58, *p* < 0.05). Series of regression analyses indicated that two handwriting performance measures (mean HPSQ time subscale and mean stroke duration) predicted 19% of planning and organizational abilities as reflected through daily functions (ADC-B) [*F*_(3, 54)_ = 38.37, β = 0.40, *p* < 0.0001].

**Conclusion:** The results support previous evidence about EF deficits as an underlying brain mechanism involved in motor coordination disorders, their significance as related to theoretical models of handwriting and daily function among DCD will be examined.

## Introduction

Despite the technological progress and the incidence of computers, handwriting is still necessary worldwide for daily tasks such as signing checks, writing a note to a family member, or writing an exam. The uniqueness of handwriting is concealed in its variations among people, being unique for each individual (Srihari et al., 2002), therefore even today, a document written by hand sometimes constitutes a criterion for procuring a job.

Whilst the generation of the written content and ideas while writing is considered the “higher level” of the writing process, the focus of the present study is on the “transcription phase” which is considered the “lower level” of writing production (Berninger and Swanson, 1994). The transcription phase relates to the processes involved in retrieving letterforms and familiar word spellings from long-term memory, strategically spelling novel words and motor planning to produce the letters by hand. Despite its being labeled “lower level,” it is evident from the literature as to the complexity of this production phase. Based on several handwriting models (e.g., Ellis, 1982; Van-Galen, 1991; Denckla and Roeltgen, 1992; Graham et al., 2006), handwriting transcription can be depicted as a hierarchically organized representation of mental motor movements. The premise of these models is that handwriting occurs because of distinct processing activities whereby the output from an earlier stage forms the input for the next stage. For example, according to Van-Galn's model, the writer first activates a lexical process which provides abstract graphemic representations which are translated into allographic code stored in a short-term motor buffer and which retrieves and releases the different motor programs required for letters writing. The parameters for executing the motor program (e.g., size of the letter) are then set, followed by neuromuscular instructions that specify the exact muscles and amount of force required producing the letter. Other researchers added that, in fact, this production ends with the process of deciding where to *place* the letter on the page (in relation to baseline and to other letters) (Denckla and Roeltgen, 1992; Graham et al., 2006). In fact, all the models describe the involvement of Executive Functions (EF) in this transcription phase with an emphasis on planning and organization in time and space.

EF is an umbrella term that encompasses high-level cognitive functions such as planning and organization, reasoning and problem solving, conceptual thought, self-correction, judgment, and decision-making (Norris and Tate, 2000; Ylvisaker and Feeney, 2002; Burgess et al., 2006).

As is seen from the handwriting models described above and further literature, varied EF components are involved in the handwriting transcription phase. EF components occupied are the ability to control attention, to maintain information in an active, quickly retrievable state (Engle, 2002), decision-making, intentional control, revising behavior (De La Paz and Graham, 1977) and planning and organization in time and space (Tseng and Cermak, 1993; Mercer, 2005; Meltzer, 2007).

In this context, Slavin et al. (1999) have indicated that handwriting may be utilized as part of neuropsychological testing, given that it is a sensitive task which can be subjected to kinematic analysis (Slavin et al., 1999).

In reality, handwriting is just one example of daily tasks, among others, which involves EF control. In fact, EF control is required from the early morning when the individual prepares for work, while organizing his home/work space and planning his daily schedule, and throughout the day. In the current study, the focus will be on daily activities which require attention control, maintaining information in an active, quickly retrievable state, decision-making and intentional control with a focus on planning and organization in time and space.

From childhood to adulthood, individuals develop their EF control through daily functions as well as their handwriting transcription skills. Thus, adults are expected to fulfill their daily tasks in a satisfactory manner, as well as to write automatically-unless suffering from some physical or mental condition that affects their handwriting performance (Longstaff and Heath, 1999).

Handwriting deficits constitute a definition appearing in a medical diagnosis manual, the DSM 4 for the diagnosis of Developmental Coordination Disorders (DCD) (Criteria's A and B) (Barnett, 2006). DCD is a marked impairment in the development of motor coordination, which significantly interferes with academic achievements or activities of daily living (ADL) [American Psychiatric Association (APA), 2000, p. 56–57]. In the past, this population was labeled as clumsy or suffering from dyspraxia or minimal brain damage. In daily functions, they are characterized by marked coordination deficits which influence their abilities to perform daily tasks such as pouring liquid, zipping, buttoning, building/fixing with small elements and organization of place/time (Rosenblum et al., 2010). The prevalence of DCD among children aged 5–11 been estimated at 6–10% (American Psychiatric Association (APA), 2000; Hamilton, 2002), while there is lack of data concerning the prevalence of DCD in adulthood (Cermak et al., 2002). Although the definition of the disability was established in 1994 [American Psychiatric Association (APA), 1994], there is still ongoing discussion in the literature about the characteristics of children with DCD, as well as the appropriate tools for DCD evaluation based on its definition (Dewey and Wilson, 2001; Green and Baird, 2005; Flapper et al., 2006). There is a considerable lack of research regarding adults with DCD despite evidence that children with DCD do not all “grow out” of their difficulties and the impact of DCD continues into adulthood (Cousins and Smyth, 2003; Kirby et al., 2008).

Despite insufficient research and evaluation tools (O'Hare and Khalid, 2002; O'Hare, 2004), deficits in handwriting transcription of children with DCD have been described in the past (Flapper et al., 2006; Rosenblum and Livneh-Zirinsky, 2008), while as far as it is known, no literature exists about the handwriting transcription features of adults with DCD. For the current study, students reported their handwriting transcription abilities in the Handwriting Proficiency Screening Questionnaire (HPSQ) (Rosenblum, 2008) and their kinematic handwriting features were evaluated with the Computerised Penmanship Evaluation Toll (ComPET) (Rosenblum et al., 2003).

In addition to evidence about difficulties in handwriting performance, deficits in EF control were also described among children with DCD (e.g., Piek et al., 2004, 2007) as well as their inferior performance of Activities of Daily Living (ADL) in comparison to controls without DCD (Dunford et al., 2005; Summers et al., 2008). However, literature related to EF and ADL among adults with DCD is scarce.

Focusing on adults with DCD, Kirby and her colleagues indicated that based on their report, 71.4% of the adults with DCD in comparison to 17.9% controls who had difficulty “*writing neatly when having to write fast*” and 55.1% (DCD) in comparison to 7.1 % (controls) who had difficulties “*organizing/finding things in your room*” (Kirby et al., 2010).

Interviews conducted among adults with DCD indicated deficits in planning and organization in space and time that influence their everyday function at home, at work and in social environments (e.g., Roffman, 2002; Rosenblum and Weintraub, 2007). Furthermore, difficulties in performing complex daily functions that involve EF with a focus on planning and organization in space and time such as driving, writing, or using technical appliances were reported among adolescents with motor difficulties (Pereira et al., 2000; Cousins and Smyth, 2003; Tal-Saban et al., 2012).

In the current study, EF as reflected in daily functions was evaluated by the Adult Developmental Co-ordination Disorders/Dyspraxia Checklist (ADC) (Kirby et al., 2010). This is a self-report scale and, in fact, expresses perceived EF as reflected through daily function.

Hence, the aims of the study were:

1. To compare the handwriting transcription features and perceived EF control as reflected through the daily function of adults with DCD to that of controls.
2. To find the relationship between handwriting transcription measures and perceived EF control through daily function in each group (DCD and controls).

The five main hypotheses of the present study were as follows:

1. Significant differences will be found between adults with DCD and controls in:
  - Handwriting legibility, performance time and related well-being, as reported by the individuals (HPSQ).
  - Temporal and spatial measures of the handwriting process, as evaluated by a computerized system (ComPET).
  - Perceived EF control as evaluated through daily function by the ADCs.
2. Significant correlations will be found between the handwriting transcription measures and perceived EF control (ADC subscales mean scores).
3. EF control as reflected through daily function (ADC) would be predicted by handwriting measures (HPSQ, ComPET).

## Materials and methods

### Participants

The sample consisted of 60 students ranging in age from 24 to 41. Thirty students with diagnosed DCD or self-reported as having motor impairments consistent with a history of DCD, and 30 age and gender matched controls.

Thirty-three percentage of the students in each group were male and 67% were female. As seen in Table 1, there were no significant difference in mean age between the groups (DCD *M* = 25.80, *SD* = 4.55 control: *M* = 26.04, *SD* = 4.66) nor for participants' years of education. All subjects were native Hebrew speakers without hearing or vision difficulties.

**Table 1:** **Background characteristics of participants in both groups**.

	**DCD group *M (SD) n = 30***		**Control group *M (SD) n = 30***	***P***
Age	25.80 (4.55)		26.04 (4.66)	NS
Years of education	13.75 (0.91)		13.75 (0.84)	NS
Years of education-mother	13.70 (3.73)		14.79 (2.93)	NS
		**Percentile %**	**Percentile %**	
Hand dominance	Right	86.7	81.3	
	Left	10	12.5	
	Mixed	3.3	–	
Handwriting difficulties-	Always	16.7	3.1	
frequency	Usually	26.7	–	
	Seldom	16.7	25.0	
	Never	40.0	68.8	

As seen in Table 1, 43% of the participants in the DCD group indicated that they always or usually have handwriting difficulties, in comparison to 3% in the control group (always). Consequently, while 69% of the participants in the control group indicated that they never have handwriting difficulties, only 40% of the DCD group indicated so.

### Instruments

#### Handwriting proficiency screening questionnaire (HPSQ) (rosenblum, 2008)

The HPSQ is a 10-item questionnaire that was developed in order to detect handwriting difficulties. The 10 items cover the most important indicators of handwriting deficiencies in the following three domains: (1) legibility (items 1, 2, 10); (2) performance time (items 3, 4, 9); and (3) physical and emotional well-being (items 5, 6, 7, 8) (Rubin and Henderson, 1982; Alston, 1983; Cornhill and Case-Smith, 1996).

In the self-report version of the HPSQ (Engel-Yeger et al., 2009), items are clear and simple to answer. For example, “Do you often erase while writing?” The items are scored on a five-point Likert scale, ranging from 1 = “never” to 5 = “always,” with higher scores indicating poorer performance. The HPSQ's content validity, internal reliability, inter-rater and test-retest reliability have been established among school-aged children (Rosenblum, 2008).

The internal reliability of the three subscales found in the current study were as follows: Legibility α = 0.79, Performance time: α = 0.78, well-being: α = 0.67, and α = 0.87 for the entire scale.

#### Computerized penmanship evaluation tool—ComPET (previously referred to as POET) (rosenblum et al., 2003)

An online-computerized handwriting evaluation was used to administer the stimuli and to collect and analyze the data. The tool includes two main parts: (1) data collection, which is language-independent and easy to use for handwriting tasks; and (2) data analysis, which has been programmed via MATLAB software toolkits.

**Figure 1:**
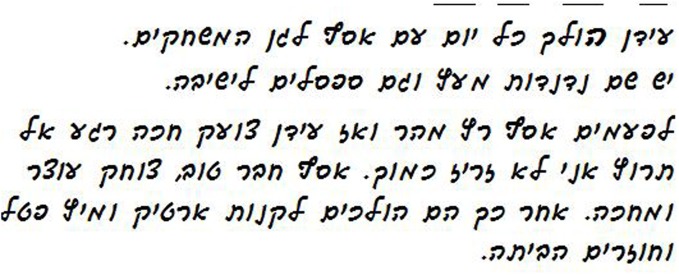
**The paragraph-copying task as appears in front of the participant (note: lines were added above words and below letters for the demonstration of Hebrew writing for the current manuscript)**.

Participants were requested to copy a paragraph set on the table in front of them, printed on paper in a Hebrew font (Gutman Yad Brush) size 20 (see Figure 1).

The present study focused on writing tasks in Hebrew. As can be seen in Figure 1, unlike Latin language writing, each word and letter in Hebrew are written separately with no connection between letters. In order to demonstrate this, the first four words are shown in Figure 1 with lines above them. Furthermore, some letters in the Hebrew alphabet are constructed from two separate, unconnected components or strokes. For example, as demonstrated in Figure 1, the second word includes four letters, of which, the first letter (HEY-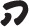), (bolded) is formed each by two separate components.

The writing task was performed on A4 lined paper affixed to the surface of a WACOM Intuos 2 [model GD 0912-12X18] *x*-*y* digitizing tablet, and using a wireless electronic inking pen [Model GP-110]. Displacement, pressure and pen tip angle were sampled at 100 Hz through a 1300 MHz Pentium (R) M laptop computer. The computerized system enables the collection of spatial, temporal, and pressure data while the subject writes. Participants were asked to copy a paragraph containing 46 words with 170 letters, a task which provides the opportunity to evaluate prolonged writing performance (see Figure 1).

Kinematic measures: Based on previous results (Rosenblum and Werner, 2006; Werner et al., 2006), we focused on the following temporal and spatial measures per written stroke:

(1) and (2) The mean stroke performance time in seconds and standard deviation of the mean stroke time in seconds.

(3) The mean stroke width in centimeters (i.e., the whole stroke width on the *x*-axis).

(4) The mean stroke height in centimeters (i.e., the whole stroke height on the *y*-axis).

#### Adult developmental co-ordination disorders/dyspraxia checklist (ADC) (kirby et al., 2010)

The ADC provides further evidence to support the diagnosis of DCD among adults, based on DSM-IV criteria. The scale includes 40 items encompassing daily activities (such as self-care, eating, dressing, playing, handwriting, driving, sociability, etc.) that require good motor coordination and executive control (attention, planning, organization in time and space) and may constitute difficulties for adults with DCD. The questionnaire consists of three subscales; the first relates to difficulties that the individual experienced as a child (subscale A-10 items) while the second (B-10 items) and third subscales (C-20 items) relate to current difficulties that the individual considers affect his performance. Each item describes a difficulty that may be experienced and the client is asked to respond on a Likert scale whether this difficulty occurs “Never” [1], “Sometimes” [2], “Frequently” [3], or “Always” [4]. The scale was arranged such that the lower the score the better the performance.

Examples of items:

Subscale A: As a child did you: Q2. Have difficulties eating without getting dirty?
Subscale B: Currently: do you have difficulties currently with the following items: Q1. Self-care tasks, such as shaving or makeup? eating with knife and fork (Q2) Finding your way around new buildings or places (Q9) etc.
Subscale C: Currently: please mark the suitable option and describe on the attached paper: Q1. Do you have difficulties with sitting still or appearing fidgety?

The ADC's reliability and validity were well-established (see Kirby et al., 2010 for more details). In the current study the internal reliability of subscales B and C was α = 0.83.

### Procedure

The ethical committee of the University of Haifa approved the study. Participants with DCD were recruited through an announcement on the bulletin boards at the University of Haifa. The announcement stated: “*If you have difficulties with handwriting, driving, buttoning and other every day activities which require motor coordination, please call*.” Based on a phone review with the responders it was then decided whether the applicant was suitable for participation in the study. Each participant from the DCD group was asked to find a friend of the same age and gender from his class and find out if he wished to participate in the study. If the subsequent telephone interview with the friend indicated that he does not have DCD, he was invited to participate as part of the control group.

All the participants who expressed their preliminary agreement to participate in the study received a document containing information about the research and signed the informed consent forms. Participants filled in the questionnaires (the demographic questionnaire and ADC) and copied a paragraph on a paper affixed to digitizer that was part of the computerized system (ComPET). If they completed the questionnaires and the handwriting task, they were offered a £10 voucher or equivalent as an honorarium.

### Data analysis

Descriptive statistics of the dependent variables were tabulated and examined.

MANOVA analyses were then used to test for group differences across the kinematic handwriting measures of the paragraph copy task and on the HPSQ and ADC subscales mean scores. Univariate ANOVA analyses were used to determine the source for the group differences.

Pearson correlations were calculated in order to investigate the associations between HPSQ subscales mean scores, handwriting performance measures, and ADC subscales mean scores in each group (DCD vs. controls).

Finally, series of hierarchical regression analyses were applied in order to determine whether kinematic handwriting measures (ComPET) and HPSQ subscales scores predict EF abilities reflected in daily function as measured by the ADC subscales scores, beyond group membership (DCD vs. typical).

## Results

1A. *Between group differences in handwriting performance as reported by the participant—The Handwriting Proficiency Screening Questionnaire (HPSQ):*

The MANOVA yielded statistically significant differences between the groups for the HPSQ scores as reported by the participants, [*F*_(3, 55)_ = 7.23, *p* < 0.0001, η^2^ = 0.28]. As presented in Table 2, the subsequent univariate ANOVA analyses revealed that the significance was due to differences between the groups in the three handwriting performance subscales (Legibility, performance time and physical and emotional well-being).

**Table 2:** **A comparison of the Handwriting Proficiency Screening Questionnaire (HPSQ) subscales scores in both groups**.

	**DCD group *M (SD) n* = 30**	**Control group *M (SD) n* = 30**	***F***_(3, 57)_	***P***	**η^2^**
Legibility	2.74 (0.98)	1.90 (0.84)	12.69	0.001	0.18
Performance time	2.69 (1.00)	1.75 (0.68)	17.44	<0.0001	0.23
Well being	2.32 (0.75)	1.67 (0.69)	12.07	0.001	0.17

1B. *Between group differences in handwriting kinematic measures:*

The MANOVA yielded statistically significant differences between the groups for the paragraph writing task across the spatial and temporal measures, [*F*_(4, 57)_ = 8.09, *p* < 0.0001, η^2^ = 0.36]. As presented in Table 3, the subsequent univariate ANOVA analyses revealed that the significance was due to differences between the groups in each of the four handwriting measures.

**Table 3:** **A comparison of the temporal and spatial measures per strokes as written in the paragraph copy task (ComPET) in both groups**.

	**DCD group *M (SD) n* = 30**	**Control group *M (SD) n* = 30**	***F*_(4, 57)_**	***P***	**η^2^**
Mean stroke duration	0.247 ± 0.096	0.199 ± 0.068	5.18	0.026	0.08
Mean stroke duration standard deviation	0.408 ± 0.273	0.270 ± 0.129	6.54	0.013	0.10
Mean stroke width	0.278 ± 0.085	0.196 ± 0.050	21.28	<0.001	0.26
Mean stroke height	0.350 ± 0.131	0.288 ± 0.067	5.51	0.022	0.08

1C. *Between groups differences in the Adult Developmental Coordination Disorders /Dyspraxia Checklist (ADC):*

Table 4 presents the means and standard deviations for the three subscales of the ADC. The MANOVA across all the three subscales yielded statistically significant differences between the two groups [*F*_(3, 56)_ = 28.67, *p* < 0.0001, η^2^ = 0.60]. As shown in Table 3, the subsequent univariate ANOVA analyses revealed statistically significant difference for the mean score of all the three ADC subscales:

**Table 4:** **A comparison of the Adult Developmental Coordination Disorders/Dyspraxia Checklist (ADC) subscales scores in both groups**.

	**DCD group *M (SD) n* = 30**	**Control group *M (SD) n* = 30**	***F*_(3, 56)_**	***P***	**η^2^**
A. As a child (10 items)	2.47 (0.55)	1.45 (0.52)	53.22	<0.0001	0.48
B. Currently (10 items)	2.08 (0.52)	1.28 (0.25)	80.63	<0.0001	0.58
C. Currently (20 items)	2.40 (0.49)	1.51 (0.26)	50.31	<0.0001	0.49

2. *Correlations between HPSQ subscales mean scores, handwriting kinematic measures and ADC subscales mean scores in each group (DCD vs. controls):*

No significant correlations were found between the handwriting kinematic spatial measures (i.e., stroke width and height) or for the ADC—A subscale (as a child).

As presented in Table 5, significant medium correlations were found in the DCD group between mean stroke duration and HPSQ time (*p* = 0.41) and HPSQ Well-being subscales scores (*r* = 0.41^*^, 0.51, *p* < 0.05, *p* = respectively), as well as with the ADCD-B subscale score (*r* = 0.47, *P* < 0.001). Furthermore, significant medium correlation was found between the HPSQ time subscale and ADC-B subscale score (*r* = 0.50, *p* < 0.05).

**Table 5:**
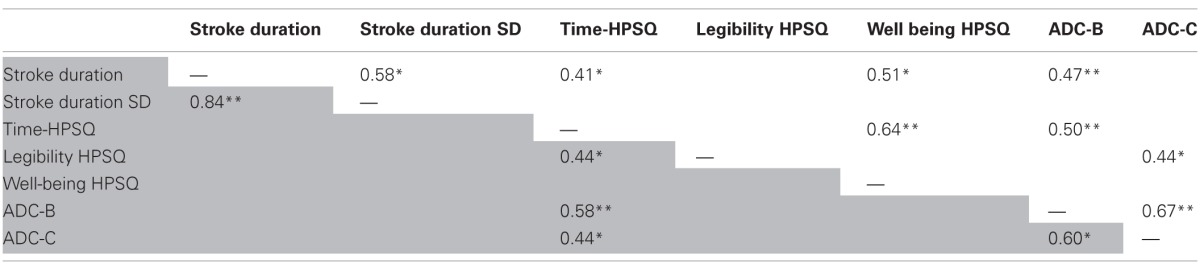
**Correlations between HPSQ subscales scores, ComPET measures and ADC subscales scores in the DCD group (upper right side of the table) and controls (lower left side of the table)**.

Among the control group, significant medium correlation was found between HPSQ time subscale score and ADC-B (*r* = 0.58, *p* < 0.001), and ADC-C subscales mean scores (*r* = 0.44, *P* < 0.05).

3. *Handwriting kinematic measures, HPSQ subscales scores as predictors of EF control through daily function (ADC).*

Initial examination presented in Table 5 indicated high significant correlations between measures of same tools: in the DCD group, between HPSQ time and HPSQ well-being subscales (*r* = 0.64, *p* < 0.0001), and in the control group, between stroke duration and stroke duration SD (*r* = 0.84, *p* < 0.001). In both groups, significant medium correlation was found between ADC-B and ADC-C subscales (DCD: *r* = 0.67, *p* < 0.0001 Control; *r* = 0.60, *p* < 0.05).

Based on those results, only one measure of each tool (HPSQ, ADC) was included in the following regression analysis.

In the first hierarchical regression, ADC-B was entered as a dependent variable in order to determine whether mean stroke duration and HPSQ time subscale score predict EF as manifested through the ADC-B, beyond the group.

The results presented in Table 6 indicated that the group accounted for 48% of the variance of the ADC-B score [*F*_(1, 56)_ = 53.01, β = 0.70, *p* < 0.0001], while the mean stroke duration added 7.5% of prediction [*F*_(2, 55)_ = 35.11, β = 0.61, *p* < 0.05] and the HPSQ time subscale added 12% to the prediction of the ADC-B mean score [*F*_(3, 54)_ = 38.37, β = 0.40, *p* < 0.0001]. As a whole, those two handwriting performance measures accounted for 19% of the variance of the ADC-B subscale score, above group membership.

**Table 6:** **Predicting perceived EF control as reflected through daily function (ADC-B subscale score) by handwriting kinematic measure and the HPSQ time subscale score**.

	**Model 1**			**Model 2**			**Model 3**		
**Variable**	***B***	**SE B**	**β**	***B***	**SE B**	**β**	***B***	**SE B**	**β**
Group	0.80	0.11	0.69^***^	0.70	0.11	0.61^***^	0.51	0.10	0.44^***^
Mean stroke duration				1.89	0.62	0.28	1.31	0.54	0.20^*^
Time subscale HPSQ mean score							0.24	0.05	0.40^***^
R^2^	48			56			68		
F change in R	53.01^***^			9.33^*^			20.69^***^		

* p < 0.05;

*** p < 0.0001.

## Discussion

The present study represents one step in addressing the challenges of understanding the functional characteristics of students who confront with DCD in their daily lives, while focusing on certain activity characteristics (handwriting) and EF control as reflected through their daily function. The literature about the functional features of this population is scarce despite the insight that the symptoms continue into adulthood (Cousins and Smyth, 2003; Kirby et al., 2008). The uniqueness of this study lies in the fact that both handwriting and EF control were assessed through self-report while for handwriting, objective measures of the process validated the self-report measures.

In reply to the question whether they deal with handwriting difficulties, 43% of the students in the DCD group indicated that they are always or are usually confronted with handwriting difficulties in comparison to 3% of the control group.

Such results indicate that handwriting deficiency, which is a meaningful and worrisome issue to parents of children with DCD (Dunford et al., 2005), continues to concern children with DCD when they grow up—during adulthood.

Indeed, results of the HPSQ indicated that students with DCD reported significantly slower performance, with significantly lower legibility. Consequently, their well-being related to handwriting performance as reflected through the HPSQ questionnaire was significantly lower in comparison to that of the controls. Their reports related to the pace of their performance were further validated by the handwriting process measures as supplied by the ComPET. Both mean stroke duration and the standard deviation of the stroke duration were significantly higher among the students with DCD.

Few studies focused on handwriting among children with DCD indeed indicated significantly longer performance time and less temporal and spatial consistency among those children, while writing (Rosenblum and Livneh-Zirinsky, 2008; Chang and Yu, 2010).

Furthermore, the current study results are in line with previous findings about differences between children with DCD and controls in processing speed (Smyth and Glencross, 1986; Dellen and Geuze, 2006), reaction time and overall timing abilities (Rosenblum and Regev, 2013). Such differences were especially marked in tasks involving both planning and execution (Henderson et al., 2006). Further studies are required in order to find whether adults with DCD are also characterized by timing deficits, and whether those deficits could be a manifestation of both cognitive and motor function deficits that co-occur in children with DCD (Kaplan et al., 2001; Hamilton, 2002; Mandich et al., 2003a; Pitcher et al., 2003; Visser, 2003).

Besides significant differences in temporal measures, the results of the current study indicate that compared to that of controls, the written product of students with DCD was significantly less legible as reflected through the legibility subscale of the HPSQ, and their written strokes were significantly higher and wider (ComPET).

Developmentally, it has been established that as a child grows, there is a decrease in letter size (Blote and Hamstra-Bletz, 1991; Lachter, 2006). This decrease is the result of more developed motor control in the distal areas of the hand and wrist, enabling the performance of hierarchical and sequential smaller movements (Thomassen and Teulings, 1985). Based on the literature about their motor coordination deficits (Kirby et al., 2010), it seems that students with DCD do not develop the appropriate control in the writing tool which will enable them to reduce letter size and produce a legible text, as done by controls. G.E described it in his words:

“My handwriting is very untidy. I have difficulty reading what I have written; I need to go over it again and again to understand what I wrote”

Summing the temporal and spatial characteristics of the handwriting of students with DCD facilitates the assumption that similar to children, their handwriting production is not automatic. Handwriting production in the transcription phase still required a process of thinking about the size, form, and direction of the letters, tending to write more slowly, less flowing and in larger letters (Wann, 1986; Berninger, 1991; Smits-Engelsman et al., 1994).

Latash (1998) indicated that automatic handwriting movements increase efficacy and reduce redundancy (Latash, 1998). The more skilled and automatic the handwriting act, the less variability there will be in temporal (performance time), and spatial (length, height, width) measures, and greater consistency will be evident (Smits-Engelsman and Van Galen, 1997).

The current results indicated that unlike the norm among people in their age, students with DCD have not acquired the ability to produce the written words in an automatic manner (Dixon et al., 1993; Srihari et al., 2002). These deficits in the transcription phase may interfere with their abilities to be available to the higher level of producing the written content (Berninger and Swanson, 1994), as they need to invest extra energy in the writing production on the paper.

There is no doubt that those handwriting deficits are meaningful to students in academia, as D.G remarked:

I can't listen and write at the same time. Sometimes I fall asleep during the lesson because I can't concentrate. If I write and listen at the same time, the writing will be very untidy.

Some explanations to the underlying mechanism behind function including handwriting were often related to children with DCD. Visio spatial and kinesthetic processing deficits were found to be connected to their functioning (Coleman et al., 2001; Wilson and McKenzie, 1998; Ameratunga et al., 2004; Piek and Pitcher, 2004). Their timing deficits may be associated with cerebellar function deficits (Lundy-Ekman et al., 1991). It was recently found that children with DCD demonstrated under-activation in cerebellar–parietal and cerebellar–prefrontal networks and in brain regions associated with visual-spatial learning (Zwicker et al., 2010).

Hence, poorer visuo-spatial short-term memory and problems in processing and storing temporal and spatial information in children with DCD as reflected through handwriting performance may underpin learning and daily function difficulties (Alloway et al., 2008) among adults with DCD as well.

Indeed, in addition to their handwriting deficits, results indicated that students with DCD were significantly different in their EF as reflected through their daily function. The groups differed in mean scores of all the three subscales of the ADC.

These results support previous results about the significant differences between children with DCD and controls in EF of organization and decision-making (Alizadeh and Zahedipour, 2005) or working memory (Piek et al., 2004). In a study conducted among adults, Kirby and her colleagues found that 52% of students with DCD aged 16–25 showed a personal weakness in EF (Kirby et al., 2008). These results are also in line with previous findings about limitations in daily life activities participation found among adults with DCD (Mandich et al., 2003b).

D.A described the manifestations in his daily function that, in fact, link timing abilities and EF deficits:

“It also happens that I forget things that I have to do, and I fail in planning my time. Sometimes I do more than I have to do, or I don't understand the instructions and have to do the same thing again. Or I don't get how much time it will take me to complete certain things, it makes me sad, frustrated and insecure”

His description supports the current findings about the significant medium correlations found between the mean score of the HPSQ time subscale and EF as reflected through daily performance (ADC-B-C), in the DCD group between the HPSQ time subscale with the ADC-B (*r* = 0.47^**^), while in the control group–between the HPSQ time subscale and both ADC-B (*r* = 0.58^**^) and ADC-C (*r* = 0.44^*^).

These results indicated not only that time deficits do not disappear in adulthood among people with DCD but that there are also clear relationships between handwriting performance time and daily function abilities.

In fact, the results of the regression give support to this link, while indicating that mean stroke duration and the HPSQ time subscale accounted for 19% of the variance of the ADC-B subscale score, above group membership.

These findings are in accordance with models of handwriting transcription that indicated the involvement of EF in the writing production, with an emphasis on planning and organization in time and space (e.g., Ellis, 1982; Van-Galen, 1991; Denckla and Roeltgen, 1992; Graham et al., 2006). Such results indicate that handwriting is indeed a complex human activity that may serve as a sensitive measure for EF as reflected in daily function in varied activities.

Both deficits in handwriting production and daily function found among students with DCD involve EF and reflect the importance of considering EF components such as attention planning and organization, reasoning and problem solving, conceptual thought, self-correction, judgment, and decision-making among adults with DCD both in evaluation and in intervention processes with this population.

Their written product and their handwriting process characteristics may be the manifestation of deficits in planning and organization in daily function. Although D. A. indicated that, “*There is no doubt that when I was young I was much more frustrated and inefficient. As years go by, you become more efficient and planned,”* longitudinal studies are required in order to find whether changes occurs in handwriting abilities and EF control as reflected through daily function from childhood to adulthood among participants with DCD.

The findings of the current study should be regarded as preliminary and still need to be confirmed in future studies with larger cohorts of patients concerning their validity, reliability, and sensitivity. However, taken together, the findings suggest that combining self-report data with objective measures of handwriting performance is, indeed, an ecologically valid assessment, which is sensitive to EF deficits manifested in the daily function of students with DCD. In addition to using the HPSQ and a computer-based analysis of handwriting performance may be used as an objective, simple, quick, and relatively inexpensive method for evaluating handwriting proficiency among this population. Combining these tools with the ADC may present a picture of both individuals' activity performance (handwriting) features as well as their EF abilities through their actual daily performances.

In sum, handwriting deficits may mirror more global deficits in EF required for daily function. Difficulties in daily function may cause this population frustration and secondary emotional and social implications with a direct influence on self-image (Kaplan et al., 2001; Skinner and Piek, 2001; Segal et al., 2002; Mandich et al., 2003a,b).

Hence there is importance in identifying the deficit in standardized tools, supplying knowledge to the individual about the phenomena, and finding the appropriate strategies in order to confront the problem and improve quality of life among this population.

### Conflict of interest statement

The author declares that the research was conducted in the absence of any commercial or financial relationships that could be construed as a potential conflict of interest.
